# CTCF maintains regulatory homeostasis of cancer pathways

**DOI:** 10.1186/s13059-018-1484-3

**Published:** 2018-08-07

**Authors:** Sarah J. Aitken, Ximena Ibarra-Soria, Elissavet Kentepozidou, Paul Flicek, Christine Feig, John C. Marioni, Duncan T. Odom

**Affiliations:** 1 Cancer Research UK Cambridge Institute, University of Cambridge, Li Ka Shing Centre, Robinson Way, Cambridge, CB2 0RE UK; 2 Department of Histopathology, Addenbrooke’s Hospital, Cambridge University Hospitals NHS Foundation Trust, Hills Road, Cambridge, CB2 0QQ UK; 3 European Molecular Biology Laboratory, European Bioinformatics Institute, Wellcome Genome Campus, Cambridge, CB10 1SD UK; 4 Wellcome Sanger Institute, Wellcome Genome Campus, Cambridge, CB10 1SA UK

**Keywords:** CTCF, Transcription, Hemizygosity, Cancer, Chromatin state, Chromatin architecture

## Abstract

**Background:**

CTCF binding to DNA helps partition the mammalian genome into discrete structural and regulatory domains. Complete removal of CTCF from mammalian cells causes catastrophic genome dysregulation, likely due to widespread collapse of 3D chromatin looping and alterations to inter- and intra-TAD interactions within the nucleus. In contrast, *Ctcf* hemizygous mice with lifelong reduction of CTCF expression are viable, albeit with increased cancer incidence. Here, we exploit chronic *Ctcf* hemizygosity to reveal its homeostatic roles in maintaining genome function and integrity.

**Results:**

We find that *Ctcf* hemizygous cells show modest but robust changes in almost a thousand sites of genomic CTCF occupancy; these are enriched for lower affinity binding events with weaker evolutionary conservation across the mouse lineage. Furthermore, we observe dysregulation of the expression of several hundred genes, which are concentrated in cancer-related pathways, and are caused by changes in transcriptional regulation. Chromatin structure is preserved but some loop interactions are destabilized; these are often found around differentially expressed genes and their enhancers. Importantly, the transcriptional alterations identified in vitro are recapitulated in mouse tumors and also in human cancers.

**Conclusions:**

This multi-dimensional genomic and epigenomic profiling of a *Ctcf* hemizygous mouse model system shows that chronic depletion of CTCF dysregulates steady-state gene expression by subtly altering transcriptional regulation, changes which can also be observed in primary tumors.

**Electronic supplementary material:**

The online version of this article (10.1186/s13059-018-1484-3) contains supplementary material, which is available to authorized users.

## Background

CCCTC-binding factor (CTCF) is a highly conserved nuclear phosphoprotein [1, 2], ubiquitously expressed in somatic cells [3], and responsible for diverse regulatory functions, including fine-tuning gene expression, X chromosome inactivation, imprinting, and three-dimensional (3D) chromatin organization [4–9]. The global 3D organization of the chromatin partitions the mammalian genome into discrete structural and regulatory domains [8, 10]. Chromosome architecture has multiple levels of spatial organization: megabase-scale compartments correspond to euchromatin (A) and heterochromatin (B) [11], sub-megabase regions can be defined as topologically associated domains (TADs) [10], and, at the tens of kilobases level, there exist smaller loop structures that connect *cis*-regulatory elements [12, 13]. Across all scales, CTCF is frequently present at these structural boundaries [14, 15].

Numerous studies have explored the function of complete disruption of CTCF binding, both in vivo and in vitro. At the whole embryo level, homozygous deletion of *Ctcf* is embryonically lethal [7], and genetically inducible *Ctcf* knockout in specific cell types, including oocytes [16], lymphocytes [17], neurons [18], and cardiomyocytes [19], results in organ-specific failure, characterized by aberrant enhancer–promoter interactions and transcriptional dysregulation [20]. Complementary biochemical approaches have tested the functional impact of acute depletion of CTCF in vitro by both RNAi [21, 22] and transient auxin-mediated depletion [23]. Acute depletion in mouse embryonic stem cells results in almost complete removal of CTCF from the nucleus, causing genome-wide disruption of loops and TADs whereas higher-order genomic compartmentalization is unaffected [23].

Despite strong conservation of the higher order chromatin structure, such as TADs, across tissues and individuals [14], there exists substantial inter- and intra-individual variation in the expression of *CTCF* [3], driven by both genetic heterogeneity and cell type specificity. Up to tenfold differences in both *CTCF* mRNA and protein expression have been observed across a variety of tissues [24, 25]. Since these differences in expression do not seem to affect the general organization of chromatin, it is not clear whether they have a functional impact. To address this, we sought a highly controlled system in which we could modulate *Ctcf* expression without resorting to a homozygous knockout.

Specifically, we utilized mice with hemizygous deletion of *Ctcf* [26, 27], a powerful strategy for dissecting direct regulatory targets and functional mechanisms [28]. Intriguingly, while *Ctcf* hemizygous mice develop normally, they have an increased predisposition to tumorigenesis [29], suggesting that even physiologically tolerated changes in CTCF concentration have a detrimental effect on the fitness of the organism. *CTCF* is also implicated as a haploinsufficient tumor suppressor gene in human cancers [2, 29, 30].

In contrast to germline variants, somatic missense and nonsense mutations of *CTCF* are common in human cancers [31, 32]. *CTCF* has been identified as a putative driver gene in several cancer types [33] and such loss of function is in keeping with the action of a tumor suppressor gene [2, 30]. Furthermore, reduced expression of *CTCF* mRNA in kidney cancer is strongly correlated with lower 5-year survival rates [34]. However, the precise role of *CTCF* in the initiation or progression of carcinogenesis is poorly understood.

To study the direct impact of altering *Ctcf* expression, independent of any factors that may confound human studies such as environmental exposures, we chose an in vitro model and exploited mouse embryonic fibroblasts (MEFs). Wild-type and *Ctcf* hemizygous MEFs were interrogated using a variety of functional assays to characterize differences in the molecular portraits between conditions. This well-defined model system allowed us to harvest the volume of cells needed to perform ChIP-seq, RNA-seq, proteomic, and Hi-C experiments from a single embryo at a low passage number. Our data reveal that *Ctcf* hemizygous cells show (1) modest but robust changes in genomic CTCF occupancy, (2) transcriptional dysregulation, enriched in cancer-related pathways, and (3) subtly perturbed chromatin looping interactions, enriched for differentially expressed genes.

## Results

### Chronic reduction of CTCF alters its chromatin binding

**Fig. 1:**
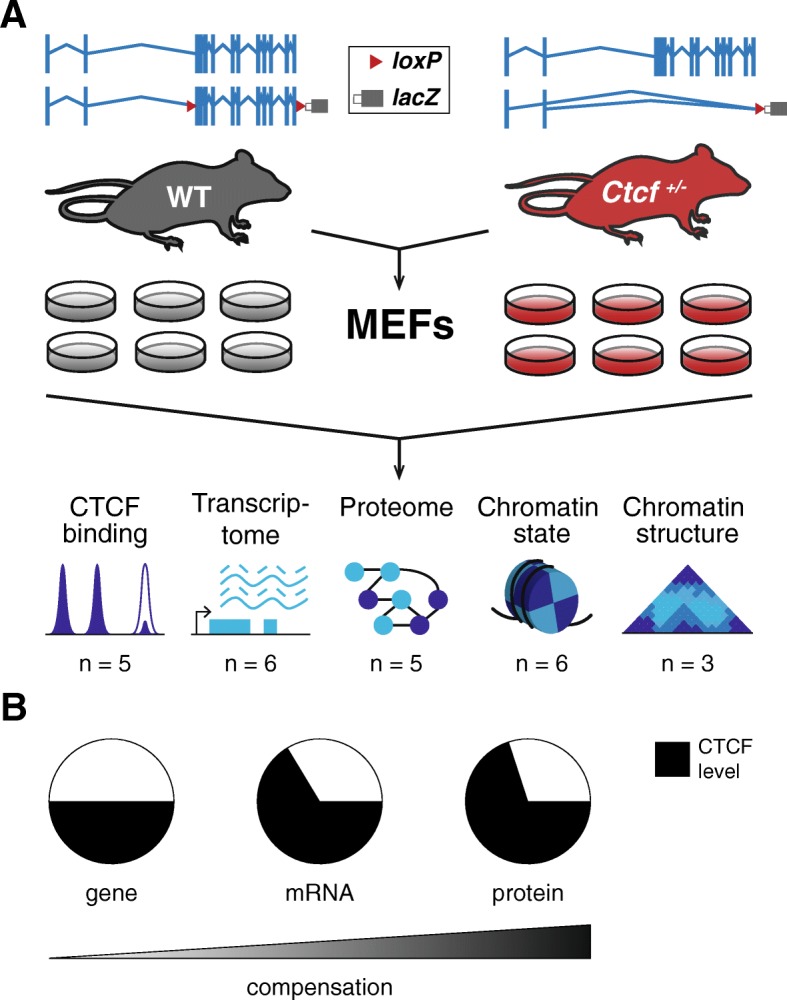
*Ctcf* hemizygosity as a model to subtly perturb nuclear homeostasis. **a** The engineered *Ctcf* locus contains *loxP* sites flanking the protein-coding exons of the gene (wild-type (*WT*), *Ctcf* ^+/+^), which can be removed using Cre recombinase (*Ctcf* ^+/−^). Mouse embryonic fibroblast (*MEF*) lines were derived from six WT and six *Ctcf* ^+/−^ littermates. Quantitative analyses of CTCF binding, transcription, proteome, chromatin state, and chromatin structure were performed in multiple biological replicates (“Methods”). **b** Validation of CTCF depletion in *Ctcf* ^+/−^ MEF cultures. Quantification of *Ctcf* deletion by qRT-PCR and quantitative western blotting experiments show that there is only partial compensation in the level of CTCF from DNA to RNA to protein

To characterize the molecular effects of altering the concentration of CTCF protein available in the nucleus, we utilized *Ctcf* hemizygous mice that carry a *lacZ* reporter in place of the coding region of *Ctcf* [27] in all cells (Fig. 1a). We derived six independent lines of embryonic fibroblasts from mice carrying a deletion of one *Ctcf* allele (*Ctcf* ^*+/−*^) and six corresponding lines from *Ctcf* wild-type littermate controls (Fig. 1a). qPCR demonstrated that *Ctcf* hemizygous MEFs had a 37% reduction (two-tailed *t*-test, *p* = 1.58 × 10^− 6^; Fig. 1b, Additional file 1) in *Ctcf* mRNA compared to wild-type. In turn, quantitative western blotting showed a 27% reduction (two tailed *t*-test, *p* = 8.731 × 10^− 5^; Fig. 1b, Additional file 1) in CTCF protein level versus wild-type cells. Thus, although there is partial compensation at both the mRNA and protein levels, there is a consistently lower concentration of CTCF in hemizygous mouse cells. We used these 12 independent embryonic fibroblast lines to generate multiple biological replicates for diverse functional experiments.

**Fig. 2:**
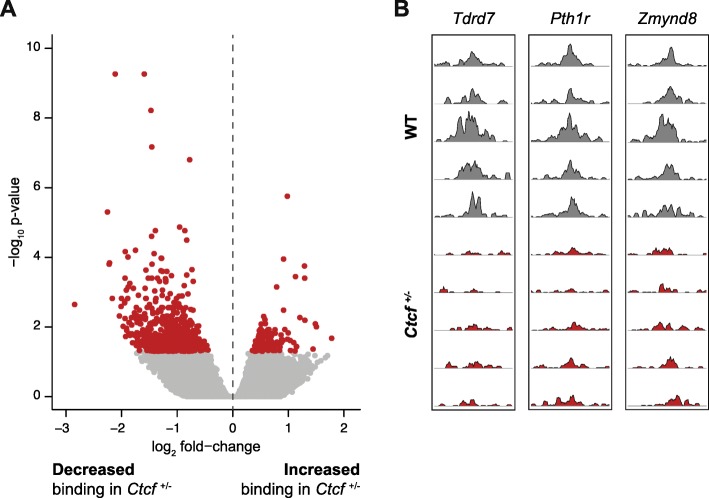
*Ctcf* hemizygosity results in altered chromatin binding. **a** Differential binding analysis identified 787 CTCF binding sites differentially occupied between *Ctcf* hemizygous and wild-type MEFs, most of which show reduced genomic occupancy in the *Ctcf* ^+/−^ MEFs. Significant changes are shown in *red* (FDR < 5%). **b** Example genome tracks showing highly consistent loss of CTCF binding at three genomic loci overlapping the genes indicated at the *top*. Data are shown for the five biological replicates that passed quality control, normalized to account for sequencing depth differences. *WT* wild-type

We first assessed the impact of hemizygosity on CTCF occupancy using chromatin immunoprecipitation followed by sequencing (ChIP-seq). We identified 42,336 loci directly occupied by CTCF, 787 of which were significantly differentially bound (false discovery rate (FDR) < 5%; Additional file 2) between the two genotypes (Fig. 2a). Of these, 79% were less strongly bound in the *Ctcf* ^+/−^ MEFs. The changes in occupancy between the genotypes were generally small but highly reproducible among independent samples (Fig. 2b). Thus, reduced availability of CTCF in embryonic fibroblasts leads to its depletion at a very specific subset of genomic sites.

### Differentially bound CTCF binding sites have distinct genomic features

**Fig. 3:**
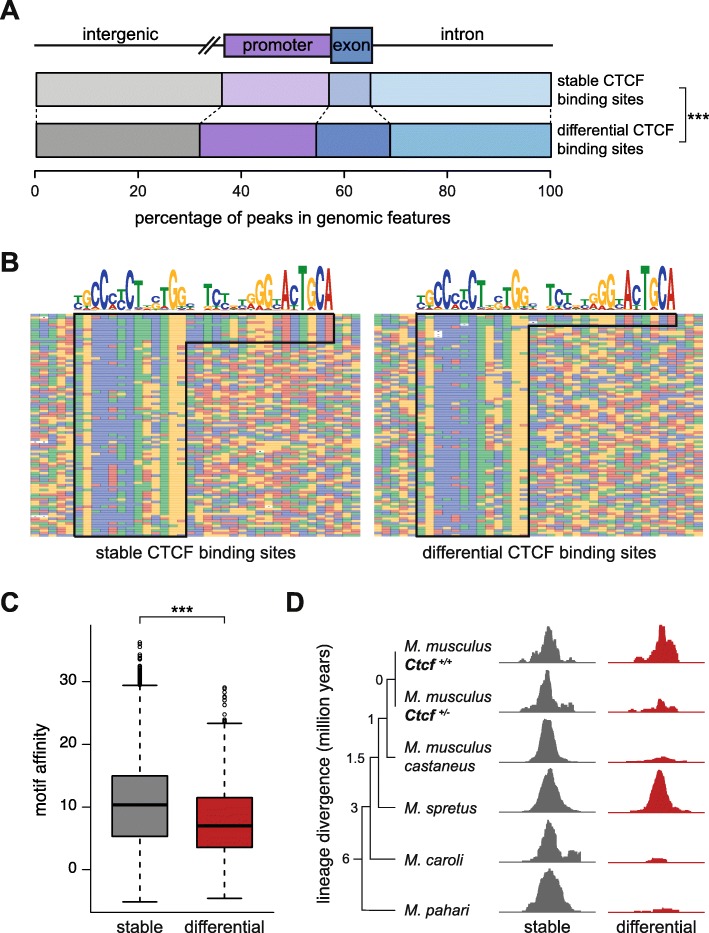
Differentially bound CTCF loci are found near genes, occur in shorter motifs, and have lower binding affinity and evolutionary conservation. **a** Differential CTCF binding sites were significantly enriched within promoters and gene bodies compared to stable CTCF binding sites (chi-square test, *p* = 4.9 × 10^− 10^). **b** Stable CTCF peaks had a higher proportion of the longer (~ 33 bp) motif word compared to the differential sites. Multiple alignments of a randomly chosen subset of a hundred CTCF binding sites that are either stable or differential are shown. Each position in the alignment is colored corresponding to the nucleotide present, following the color scheme used in the CTCF motif logo shown at the *top*. **c** Binding sites susceptible to reduced CTCF concentration have significantly lower motif affinity (Mann-Whitney test, *p* < 2.2 × 10^− 16^). **d** Regions bound by CTCF across the mouse lineage are less sensitive to *Ctcf* hemizygosity. Example tracks are shown of a stable CTCF binding site that is conserved in five species of mice, compared to a differential site that is found in only a subset of the species, *M.* = *Mus*. (*M. musculus* chr6:120,736,800 for the stable site and chr2:31,887,060 for the differential site). *** *p* value < 0.001

Genomic locations sensitive to subtle and chronic CTCF reduction shared a number of features. First, most (68%) of the differentially bound sites overlapped annotated genes or their promoters (defined as 5 kb upstream of the transcription start site), a fraction that is significantly enriched compared to genome-wide CTCF occupancy (chi-square test, *p* = 4.9 × 10^− 10^; Fig. 3a). Second, CTCF can bind motif instances of either ~ 20 or ~ 33 bases [22]; we found that differentially bound CTCF sites were significantly depleted of longer words (hypergeometric test, *p* = 1.53 × 10^− 11^; Fig. 3b). Previous studies have shown that binding sites with shorter motifs have lower average binding affinity [22]. Consistent with this, CTCF sites perturbed by hemizygosity had motifs of lower affinity when compared to all CTCF bound regions (Mann-Whitney test, *p* < 2.2 × 10^− 16^; Fig. 3c). And third, by comparing with the ~ 11,000 CTCF sites conserved across five species of mice [35], we discovered that differentially bound CTCF sites were depleted of these conserved binding events (hypergeometric test, 2.55 × 10^− 6^; Fig. 3d). In other words, CTCF binding sites stable across the murine lineage are resistant to chronically reduced levels of CTCF.

In conclusion, our data reveal a set of regions preferentially found near genes which show reproducible, quantitative changes in CTCF occupancy, have common motif characteristics, and are enriched for lineage-specific CTCF binding.

### *Ctcf* hemizygosity alters transcription of cancer pathways

**Fig. 4:**
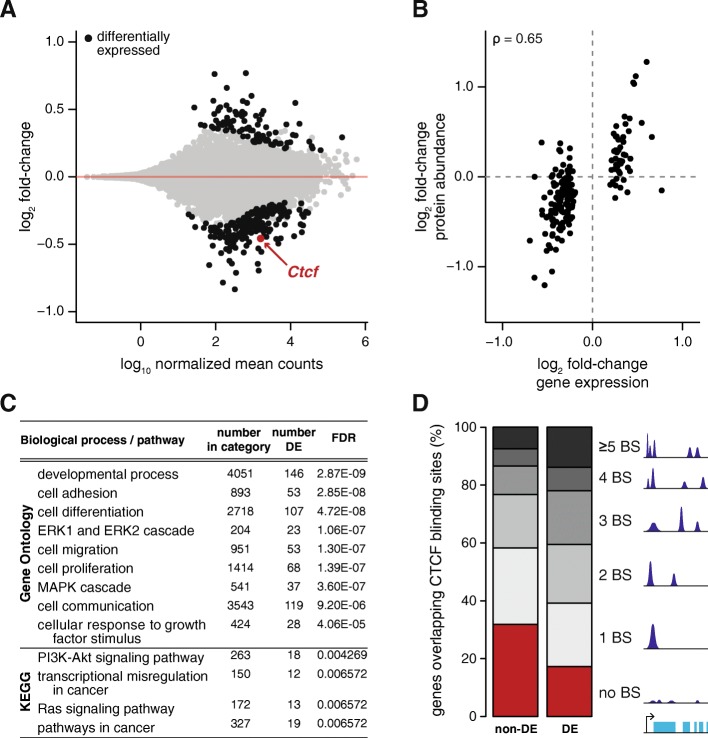
CTCF depletion dysregulates oncogenic pathways. **a** Nearly 300 genes are differentially expressed between wild-type and *Ctcf*^*+/−*^ cells; significant changes are shown in *black* (FDR < 5%). **b** Transcriptional changes (x-axis) were highly correlated to corresponding changes at the protein level (y-axis); Spearman’s correlation coefficient is noted in the *top left corner*. Of all genes from **a**, 85% had concordant fold-change estimates in the proteomics dataset. **c** Gene set enrichment analysis performed on the differentially expressed (*DE*) genes highlights dysregulation of cancer related pathways. Representative significantly enriched terms from the Gene Ontology and KEGG pathways are shown. **d** Differentially expressed genes are strongly enriched for having higher numbers of CTCF binding sites (*BS*) than genes with stable expression, in their gene bodies or flanking 5 kb

To determine what impact changes in CTCF binding had on the global transcriptome, we sequenced total RNA from six biological replicates of both genotypes. Confirming the qPCR results, hemizygosity resulted in a significant reduction in *Ctcf* expression (*p* = 2.4 × 10^− 7^; “Methods”). Consistent with the differences in CTCF occupancy, transcriptional changes were subtle: differential gene expression analysis identified 296 dysregulated genes (FDR < 5%; Additional file 3), 69% of which had reduced expression in *Ctcf* ^+/−^ MEFs (Fig. 4a).

The changes observed in the transcriptome propagated to the protein level, as shown by comparison to the proteomes of the wild-type and hemizygous cells obtained using tandem mass tag (TMT) mass spectrometry (Additional file 4). For the differentially expressed genes, the transcriptional and proteomic changes in *Ctcf* hemizygous cells were highly correlated (Spearman’s rho = 0.65, *p* < 2.2 × 10^− 16^; Fig. 4b). Indeed, 85% had fold-change estimates concordant between RNA transcription and protein expression.

Gene set enrichment analysis revealed that CTCF-dependent transcripts were strongly enriched for processes related to cell differentiation, proliferation, death, migration, adhesion, angiogenesis, and protein phosphorylation. The MAPK, ERK1/2, and Ras signaling pathways also showed an excess of dysregulated transcripts (Fig. 4c; Additional file 3). Consistent with these results, analysis of KEGG pathways revealed that *Ctcf* hemizygosity resulted in perturbation of cancer-related pathways (Fig. 4c, Additional file 3).

Finally, we asked whether these gene expression changes could be caused directly by altered CTCF binding. We observed that few differentially bound CTCF sites overlapped, or were in close proximity to, the genes with altered expression. However, there was a strong tendency for differentially expressed genes to be associated with higher numbers of CTCF binding sites (Fig. 4d), even if these were stable. For example, 83% of all dysregulated genes (± 5 kb) overlapped at least one CTCF binding site, in contrast to only 68% of stable genes (hypergeometric test, *p* = 1.32 × 10^− 8^). Further, whereas only 23% of stable genes overlapped with three or more CTCF bound sites each, 41% of all differentially expressed genes did (Fig. 4d). Thus, the set of genes dysregulated in *Ctcf* hemizygous cells are strongly enriched for CTCF binding sites, suggesting subtle additive effects regulate nearby gene transcription.

### Gene expression changes correspond with altered looping interactions

**Fig. 5:**
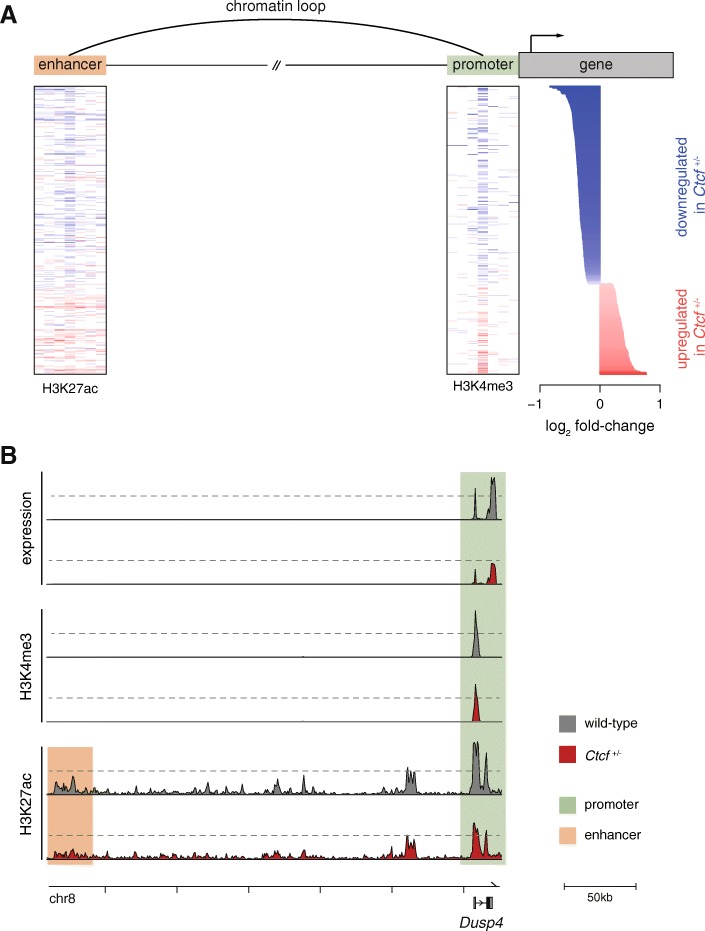
Transcriptional perturbations arise from regulatory changes in the nuclear genome. **a** Changes in expression of dysregulated genes are accompanied by changes in the activity of their proximal promoters, as well as enhancers linked via chromatin loops. On the *right* the expression differences between wild-type and *Ctcf* ^+/−^ cells are shown, ordered by increasing fold change; genes expressed at lower levels in the hemizygous cells are in *blue*, whereas those expressed higher are in *red*. To the *left*, a heatmap of the difference in mean abundance of H3K4me3 occupancy is shown. Each column is a 5 kb window, extending 17.5 kb up- and downstream of each gene’s transcription start site, which is in the center. On the *far left*, an equivalent heatmap for the difference in occupancy of H3K27ac, centered at the midpoint of the peak. Gene–enhancer pairs were inferred from significant interactions identified from Hi-C data and thus elements can be separated by large distances. For each gene, the enhancer with most regulatory potential is shown (“Methods”). The same color scale is used throughout. **b** Transcriptional changes are accompanied by concordant changes in the activity of their regulatory elements. In *Ctcf* hemizygous cells there was reduced gene expression of *Dusp4*, lower occupancy of promoter marks (H3K4me3 and H3K27ac), and reduced binding at the associated enhancer element (*shaded boxes*). Two representative replicates of equivalent sequencing depth are shown for each dataset

Steady-state transcription can be altered either by changes in transcript stability or by differences in transcriptional regulation. We examined whether the promoters of CTCF-dependent genes showed corresponding changes in transcriptional initiation, reflected as changes in H3K4me3 and H3K27ac occupancy. Both of these histone modifications are associated with an open chromatin state, permissive of active transcription [36–39] and their occupancy levels at the transcription start site are positively correlated with gene expression [36]. The vast majority (> 80%) of the CTCF-dependent genes had concordant promoter and transcriptional changes (Fig. 5a). Thus, most gene expression differences apparently arise from CTCF-mediated alterations to transcriptional initiation.

Gene expression can be controlled by looping interactions between regulatory elements, mediated by CTCF [40–45]. To determine the effects of hemizygosity on chromatin architecture we performed nuclear Hi-C experiments in three biological replicates of wild-type and *Ctcf* ^+/−^ MEFs. First, we inspected the global-scale interaction profiles in both genotypes using 100 kb windows covering the whole genome. Consistent with recent studies showing that acute total depletion of CTCF results only in modest effects on large-scale chromatin interactions [23], we found that 95% of all windows were unaffected by reduced CTCF, showing a correlation coefficient of 0.9 or higher between the genotypes.

To explore whether fine-scale chromatin organization is affected by reduced levels of CTCF, we merged all replicates to increase the resolution of our data and identified pairs of loci that interact more often than expected by chance. We then compared the intensity of such interactions in the wild-type and hemizygous cells and generated a ranked list. Looping interactions that involved a dysregulated gene or a differentially bound CTCF site tended to rank higher and were significantly enriched at the top of the list (Wilcoxon signed rank test, *p* = 0.017 for genes and *p* = 2.72 × 10^− 6^ for CTCF sites). Thus, many of the transcriptional changes we observed may indeed be the result of changes in distal regulatory elements mediated by looping interactions.

We reasoned that these loops were likely to connect differentially expressed genes to distal enhancers that showed concordant changes in activity. We defined putative enhancers as sites occupied by H3K27ac but lacking H3K4me3 [37, 38] and identified 73,670 loci with this epigenetic profile. We then collected the subset of enhancers associated with a dysregulated gene via a looping interaction (“Methods”) and compared the fold-change between the wild-type and *Ctcf* hemizygous cells. Of these enhancer–gene pairs, 75% showed concordant changes between gene expression and enhancer activity (Fig. 5a). Bulk analysis of enhancer changes would not have identified these connections since direct comparison of *Ctcf* hemizygous and wild-type cells found almost no enhancer differences, with only 127 (0.2%) being significantly differentially bound (FDR < 5%; Additional file 2). Thus, the transcriptional changes observed in the hemizygous cells are likely to result from altered transcriptional regulation mechanisms that involve both promoters and distal enhancers.

For instance, *Dusp4* is an ERK phosphatase that acts as negative regulator of the MAPK pathway [46] and its gene expression is reduced in *Ctcf* hemizygous cells compared with controls (*p* = 0.026; Fig. 5b). The promoter activity of *Dusp4*, measured by chromatin co-occupancy by H3K4me3 and H3K27ac, was also reduced in *Ctcf* hemizygous cells, as was its candidate enhancer identified by looping interactions in our Hi-C experiments (Fig. 5b). Because of its action as a negative regulator, reduced expression of *Dusp4* causes aberrant MAPK activation [47]; this pathway plays a critical role in the initiation and progression of cancer [48].

### Altered gene expression patterns are recapitulated in mouse and human tumors

In order to assess the relevance of our findings to the process of tumorigenesis in vivo*,* we asked whether CTCF-dependent cancer pathways were also activated in the transcriptomes of primary mouse and human tumors. Notably, *CTCF* is detected as a mutational driver in uterine and breast carcinomas, in which most (68%) variants are truncating mutations [31, 32]; therefore, our *Ctcf* hemizygous model is a good parallel to this human molecular phenotype.

**Fig. 6:**
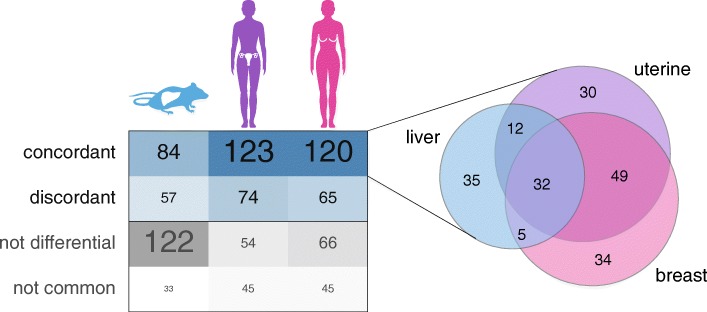
Concordant gene alterations in diverse murine and human tumors. The comparison of the set of CTCF-dependent genes and those differentially expressed in mouse liver tumors or in human uterine and breast tumors revealed a large overlap. The majority of these changed in the same direction (concordant) in the *Ctcf* hemizygous MEFs and the tumor samples. Additionally, the set of genes unique to MEFs is indicated (not differential) and those that were either not expressed or did not have a one-to-one ortholog in the human genome (not common). The concordant gene changes across these diverse tumors are highly overlapping, as seen in the Venn diagram

First, we generated a novel cohort of 25 primary liver tumors that spontaneously occurred during aging of C3H mice [49]. We then analyzed their total RNA transcriptome (“Methods”) with a set of normal liver controls. We compared the set of differentially expressed genes in these mouse liver tumors (“Methods”; Additional file 5) with the genes perturbed by *Ctcf* hemizygosity in MEFs and found that nearly half (47.6%) of the latter were also differentially expressed in the tumors. The majority (60%) showed concordant fold changes (Fig. 6), indicating that a large proportion of the up- and down-regulated genes in the *Ctcf* ^+/−^ cells were also up- and down-regulated, respectively, in the mouse liver tumors. Notably, these concordantly altered genes retained strong enrichment for cancer-related functional terms and pathways (Additional file 5).

We next asked whether the molecular pathways perturbed by *Ctcf* hemizygosity in mouse embryonic fibroblasts were similarly perturbed in human tumors. We identified 104 uterine and 19 breast human tumor samples from The Cancer Genome Atlas with deleterious (missense, frameshift, or stop gained) mutations in at least one allele of *CTCF*. To compare the gene expression profiles across species, we restricted our analyses to those genes that are one-to-one orthologs (“Methods”). For both the uterine and breast cancer datasets, we observed a large overlap (~ 75%) between the set of differentially expressed genes in *Ctcf* hemizygous MEFs and those altered in human tumors. From these, around 65% showed concordant changes across all datasets (Fig. 6), supporting a common signature of transcriptional alterations upon the loss of one functional copy of *CTCF*.

In sum, our data indicate that a small reduction in the concentration of CTCF can significantly perturb the expression of hundreds of transcripts required for normal cellular homeostasis, as evidenced by their dysregulation in a diversity of mouse and human tumors.

## Discussion

Complete removal of CTCF has catastrophic effects caused by massive dysregulation of the 3D genome [19, 22, 50, 51]. We have used *Ctcf* hemizygosity as a powerful model system to compare how transcription and genome organization in otherwise identical cells adapt to differing concentrations of CTCF. This model closely approximates the normal physiological variation of CTCF levels across tissues, without the confounding effects that arise from cell-specific *trans* environments.

Our data strongly suggest that mammalian cells can compensate for fluctuations in intracellular CTCF concentration at each level, from DNA to protein. In MEFs, removal of 50% of the *Ctcf* gene content causes a 37% reduction in mRNA expression, leading to a 27% reduction in CTCF protein, which results in only a 2% difference in genomic occupancy. The homeostatic and functional buffering observed in our model system offers a clear explanation for how tissues that have highly variable levels of CTCF expression [24, 25, 52] nevertheless preserve CTCF genomic occupancy levels.

Our data further indicate that submegabase-scale chromatin structures are also robust to variation in the amount of CTCF available in the nucleus. Recent studies have shown that CTCF is dispensable for the establishment of the A and B compartments, but necessary for the proper insulation of TADs and the integrity of looping interactions [23]. We did not observe any changes in the structure or insulation of TADs in the *Ctcf* hemizygous cells (data not shown), consistent with the high conservation of TADs observed across tissues.

The controlled reduction of CTCF expression in hemizygous cells revealed, however, reproducible changes to the nuclear environment, thus providing insights into the inherent functions of this essential protein. We identified almost a thousand loci directly bound by CTCF that showed reproducible quantitative changes in their genomic occupancy. These were accompanied by alterations in several hundred genes, which in turn affected the corresponding protein abundances. Because the promoters of these genes were differentially deployed between the two genotypes, these transcriptional changes arose from alterations in nuclear homeostasis, not differences in transcript stability in the cytoplasm. Near these dysregulated genes, we observed an excess of unstable fine-scale chromatin interactions, as well as enhancers connected via loops that showed altered activity. Therefore, in contrast to high-order chromatin structures, which are indifferent to fluctuations in CTCF concentration, fine-scale genome organization is more sensitive, and these alterations impact the regulatory landscape leading to a perturbed functional state.

The transcriptional alterations we observe may be the result of increased variability in the expression of these genes. Indeed, the loss of promoter–enhancer interactions due to knockdown of CTCF or the deletion of its binding sites can result in increased cell-to-cell variability in gene expression [53]. Whether ultimately due to increased cell-to-cell variability or to gene-specific changes occurring uniformly across cells, reduction in CTCF results in highly reproducible changes in the cells’ epigenome and transcriptome. These gene expression changes were found disproportionately among cancer-related pathways and, consistently, a large proportion of these genes are dysregulated in the transcriptomes of mouse and human tumors from diverse origins.

## Conclusions

Our data support the hypothesis that, although mammalian cells are tolerant to a reduced concentration of CTCF, there is specific dysregulation of oncogenic pathways that confers an increased predisposition to carcinogenesis.

## Methods

### Mouse models

All animal experimentation was carried out in accordance with the Animals (Scientific Procedures) Act 1986 (United Kingdom) and with the approval of the Cancer Research UK Cambridge Institute Animal Welfare and Ethical Review Body. All animals were maintained using standard husbandry: mice were group housed in Techniplast GM500 Mouse IVC Green Line cages in a room with 12 h light/12 h dark cycle and *ad libitum* access to water and food (LabDiet 5058). Cages contained aspen bedding and the following cage enrichments: nesting material, aspen chew stick, and cardboard tunnel.

C57BL/6J and C3H/HeOuJ mice were obtained from Charles River Laboratories. B6.C-Tg(Pgk1-cre)1Lni/CrsJ mice, referred to hereafter as Pgk-Cre, were obtained from the Jackson Laboratory. *Ctcf*^flox/flox^ mice [27] were a gift from Niels Galjart (Erasmus MC, Rotterdam).

### Mouse embryonic fibroblast cultures

Male *Ctcf*^flox/flox^ mice were crossed with *Pgk-Cre*-positive females, producing *Ctcf* hemizygous (*Ctcf* ^+/−^) offspring. The hemizygous *Ctcf* deletion was maintained on a C57BL/6J background by crossing *Ctcf* ^+/−^ males with C57BL/6J females, resulting in hemizygous (*Ctcf* ^+/−^) and wild-type (*Ctcf* ^+/+^) littermates; these embryos were used to derive mouse embryonic fibroblast (MEF) cultures.

Embryos were collected 13 days after vaginal plugs were identified (E13.5). The head and visceral organs were removed and used for genotyping with real-time PCR (Transnetyx). The remaining embryonic tissue was minced and trypsinized at 37 °C for 30 min, quenched with “MEF media” (Dulbecco’s modified Eagle medium (DMEM, Gibco) supplemented with 4.5 g/L D-glucose, L-glutamine, and pyruvate, 10% heat inactivated fetal bovine serum (FBS, Gibco), 1% amphotericin B antimycotic (Life Technologies), and 1% penicillin-streptomycin solution), and each embryo suspension was seeded into a 15 cm dish and incubated at 37 **°**C in 5% CO_2_. When confluent, cultures were split 1:3, in the absence of antibiotics from passage 2 onwards. ChIP-seq, RNA-seq, proteomics, and Hi-C experiments were all performed from a single passage 4 (P4) culture. MEF cultures for each biological replicate were expanded and harvested in pairs, one wild-type and one *Ctcf* hemizygous line at a time, to control for culture-related batch effects.

### Quantitative PCR

Total RNA was extracted from P4 MEF cultures from six biological replicates from each genotype, using QIAzol Lysis Reagent (Qiagen), according to the manufacturer’s instructions. cDNA was synthesized from RNA using a High-Capacity RNA-to-cDNA Kit (Thermo Fisher Scientific), and qPCR was performed in three technical replicates using TaqMan probes (Thermo Fisher Scientific) according to the manufacturer’s instructions. *Ctcf* mRNA levels were calculated using mean Ct values, normalized to *Gapdh* signal, for each pair of MEF cultures. Results are detailed in Additional file 1.

### Quantitative western blotting

Protein was extracted from P4 MEFs from six biological replicates from each genotype: cells were washed with ice cold PBS, lysed in radioimmunoprecipitation assay (RIPA) buffer, pulse sonicated on ice (3 × 10 s), and agitated for 30 min at 4 °C; the cell debris was pelleted by centrifugation and the supernatant was quantified using a Direct Detect Infrared Spectrometer (Merck Millipore). Total protein (20 μg) was run on a 4–12% Bis-Tris gel and transferred to a membrane using an iBlot 2 Gel Transfer Device (Thermo Fisher Scientific). The membrane was blocked using Odyssey Blocking Buffer in TBS (LI-COR Biosciences), incubated overnight with CTCF anti-rabbit antibody (Cell Signaling Technology, D31H2 XP; 1:1000) and β-actin anti-mouse antibody (Sigma, clone AC-74; 1:5000). The membrane was washed 4 × 5 min in TBS + 0.1% Tween and incubated for 45 min at room temperature with fluorescent-conjugated infra-red (LI-COR Odyssey) antibodies: goat anti-mouse antibody (1:20,000) labeled with 680 LT infrared dye (P/N 925–68070) and goat anti-rabbit antibody (1:5000) with 800 CW infrared dye (P/N 925–32211). The membrane was washed a further four times before visualization and quantification using the Odyssey CLx Imaging System. Relative CTCF abundance was calculated for each pair of MEF cultures using normalized fluorescence values, using β-actin as the loading control. Results are detailed in Additional file 1.

### ChIP-seq

MEFs for six biological replicates from each genotype were fixed in DMEM containing 1% fresh formaldehyde and incubated at room temperature for 10 min, quenched with 250 mM glycine for 10 min, and washed twice with ice cold PBS. The fixed cells were lifted off the plate, pelleted by centrifugation, and flash-frozen at −80 °C. Cross-linked cells were lysed according to published protocols [54]. Sonication was performed using a Bioruptor Plus (Diagenode) sonicator to fragment chromatin to an average length of 300 bp. The following antibodies (10 μg) were used for immunoprecipitation: CTCF (rabbit polyclonal, Merck Millipore 07–729, lot 2517762); H3K4me3 (mouse monoclonal IgG clone CMA304, Merck Millipore 05–1339, lot 2603814); H3K27ac (rabbit polyclonal IgG, Abcam 4729, lot GR244014–1). Immunoprecipitated DNA or 50 ng of input DNA was used for library preparation using the ThruPLEX DNA-Seq library preparation protocol (Rubicon Genomics, UK). Library fragment size was determined using a 2100 Bioanalyzer (Agilent). Libraries were quantified by qPCR (Kapa Biosystems). Pooled libraries were sequenced on a HiSeq4000 (Illumina) according to manufacturer’s instructions to produce single-end 50 bp reads.

### ChIP-seq data alignment and quality control

Raw sequencing reads from ChIP and input libraries were aligned to the mouse reference genome (GRCm38) using bwa 0.7.12 [55] on backtrack mode, with default options. The resulting SAM files were manipulated with SAMtools 1.3 [56]. Duplicated reads were marked with MarkDuplicates 1.139 from Picard tools (http://broadinstitute.github.io/picard).

Quality control (QC) of samples was performed using Phantompeakqualtools (https://www.encodeproject.org/software/phantompeakqualtools/) [57] and only those with a positive quality tag were used in downstream analyses; thus, we removed replicate 1 from the CTCF dataset and replicate 6 from the H3K27ac dataset. All regions within the ENCODE blacklist (http://mitra.stanford.edu/kundaje/akundaje/release/blacklists/mm9-mouse/mm9-blacklist.bed.gz) [58] were excluded; the *liftOver* function from the rtracklayer 1.34.1 [59] Bioconductor package was used to convert the coordinates to the mm10 (GRCm38) assembly. Further, any regions with high signal in our own inputs were also excluded; these were identified with the *greyListBS* function from the package GreyListChIP 1.6.0 [60], using the merged input datasets.

### Differential binding analysis of ChIP-seq data

CTCF differential binding between the two genotypes was performed with csaw 1.8.0 [61], with a window size of 15 bp, spacing of 50 bp, and a fragment length estimated from cross-correlation analysis. Duplicate reads were retained but any reads with mapping quality below 30 were ignored. We checked for evidence of global composition biases in the data, but the estimated size factors to correct for this were all very close to one. Thus, count data were normalized for efficiency biases instead. In the differential test we controlled for the batch effect from sample collection time. Windows were merged into regions if they were within 100 bp of each other, restricting the maximum width to 5 kb; this resulted in 42,336 regions. The combined *p* value for each region was computed with the Simes’ method, upweighting the highest abundance windows (peak summit). Regions with a corrected *p* value of 0.05 or lower (FDR < 5%) were considered significantly differentially bound. This yielded a set of 787 differentially bound regions, 79.4% of which were less bound in the hemizygous cells. The remaining 162 loci showed a relative enrichment in occupancy compared to the wild-type. Since ChIP-seq quantification is relative in any given sample, the loss of binding in several hundred CTCF binding sites leads to a proportional increase in sequencing reads at other bound loci. We find that these 162 regions are in general of higher affinity (Mann-Whitney test, *p* value = 9.13 × 10^− 24^) and evolutionary conservation (hypergeometric test, *p* value = 5.29 × 10^− 10^; see below), and longer in width, compared to the stable or less bound binding sites. This is consistent with the compensation expected as a result of the loss of some binding events.

To check the validity of the regions defined as peaks with the csaw method, MACS2 callpeak [62] was run with options -g mm -s 50 -q 0.01 --call-summits, using all ChIP libraries merged together along with the corresponding merged inputs. MACS2 reported 47,075 significant peaks, and these contained 97.9% of all csaw regions, verifying that the regions tested for differential binding were significant peaks.

To test for differential binding on the histone data, peaks were called with MACS2 as detailed above. For the H3K4me3 dataset we kept the option --call-summits and for the H3K27ac dataset we used instead --broad. Then, DiffBind 2.2.7 [63] was used to test for differences between the genotypes. Fragment sizes were determined from cross-correlation analyses. To count reads in peaks, we used a summit size of 200 bp for the H3K4me3 dataset and the whole peak for the H3K27ac data. We set *bRemoveDuplicates* to FALSE and *mapQCth* to 30. For differential testing, we controlled for the batch effect from sample collection time and set the options *bSubControl* and *bFullLibrarySize* to FALSE when calling *dba.analyze*; we used the edgeR method. For the H3K27ac data the analysis was performed for two mutually exclusive sets of peaks: those that overlapped an H3K4me3 peak (representing promoters) and those that did not overlap an H3K4me3 peak (putative enhancers).

Detailed results of the differential binding analyses are provided in Additional file 2. The MACS2 peak calls are provided as processed data in ArrayExpress (see below for dataset identifiers).

### Motif analysis on CTCF binding sites

To identify the motifs in the genomic loci occupied by CTCF, the 500 bp DNA sequences centered at the midpoint of the regions defined in the csaw analysis (see above) were extracted. These were then fed to the MEME-ChIP suite [64] for *de novo* motif identification and comparison to the JASPAR Vertebrates and UniPROBE Mouse databases. The most significant motif identified was the canonical CTCF motif (M1) and over 90% of all regions had at least one match. The third most significant motif identified was M2 as defined in [22]. For Fig. 3b 100 stable and 100 differential CTCF binding sites were randomly selected. We then collected the coordinates of the M1 motif from the MEME output and extracted the genomic sequences plus 20 nucleotides on both sides. For binding sites with multiple motifs we selected the one that best matched the motif consensus. The obtained sequences were aligned with MUSCLE [65] using default parameters.

### CTCF motif affinity

To quantify the affinity of each CTCF motif instance identified from our ChIP-seq data we used DeepBind [66], a deep learning algorithm that has been trained on large amounts of ChIP-seq data and can be used to score the affinity of any given sequence for the CTCF motif. The same 500 bp DNA sequences used for motif discovery (see above) were used to score their motif affinity with DeepBind v0.11, using motif D00328.018 (CTCF). Similar results were obtained if we scored only the motif sequence identified by MEME-ChIP.

### Mouse conservation analysis

To investigate whether differentially bound CTCF binding sites have different evolutionary dynamics to stable sites, C57BL/6 CTCF peaks were mapped to their orthologous regions on the genomes of four other mouse species: *Mus musculus castaneus*, *Mus spretus*, *Mus caroli*, and *Mus pahari*. This was performed using a multiple whole-genome alignment of 17 eutherian mammals [67] plus *mcast*, *mspr*, *mcar*, and *mpah* [35]. A CTCF peak was defined as conserved across all five mouse species if its orthologous locus in each species was also proven to bind CTCF based on ChIP-seq data derived from that species [35]. Significant depletion of conserved peaks in the set of differentially bound CTCF sites was tested using a hypergeometric test.

### RNA-seq

Total RNA was extracted from P4 MEF cultures from six biological replicates from each genotype, using QIAzol Lysis Reagent (Qiagen), according to the manufacturer’s instructions. DNase treatment and removal were performed using the TURBO DNA-freeTM Kit (Ambion, Life Technologies), according to the manufacturer’s instructions. RNA (1 μg) was used to generate sequencing libraries using the TruSeq Stranded Total RNA Library Prep Kit with Ribo-Zero Gold (Illumina), according to the manufacturer’s instructions. Library fragment size was determined using a 2100 Bioanalyzer (Agilent). Libraries were quantified by qPCR (Kapa Biosystems). Pooled libraries were sequenced on a HiSeq4000 according to the manufacturer’s instructions to produce paired-end 150 bp reads.

### RNA-seq data processing and analysis

RNA-seq paired-end fragments were aligned to the mouse reference genome (GRCm38) with STAR 2.5.2a [68] with options --outFilterMismatchNmax 6 --outFilterMatchNminOverLread 0.4 --outFilterScoreMinOverLread 0.4 --outFilterType BySJout --outFilterMultimapNmax 20 --alignSJoverhangMin 8 --alignSJDBoverhangMin 1 --alignIntronMin 20 --alignIntronMax 1000000 --alignMatesGapMax 1000000 --outSAMstrandField intronMotif. On average, 77% of the sequencing fragments mapped uniquely. The numbers of fragments overlapping annotated transcripts were obtained with the program featureCounts 1.5.2 [69] from the Subread package, using Ensembl’s genome annotation [70] version 84 (http://mar2016.archive.ensembl.org/Mus_musculus/Info/Index). The sequence of the *lacZ* cassette was added to the genome to corroborate the genotype of each sample.

For differential expression analysis, hidden batch effects were identified with the Bioconductor package sva 3.22.0 [71], providing known batch effects (sample collection time). We then used DESeq2 1.14.1 [72] to test for differential expression, controlling for both the known and hidden batch effects. Genes were considered significantly differentially expressed if their adjusted *p* value was lower than 0.05 (FDR < 5%).

Significantly enriched Gene Ontology terms were identified using the functions goana and kegga from the Bioconductor package limma 3.33.14 [73], with gene length as a covariate. The gene lengths supplied were obtained from the featureCounts output (see above). The reported *p* values were corrected for multiple testing by the Benjamini and Hochberg method and were considered significant if they were lower than 0.05. Detailed results of both the differential expression and gene set enrichment analyses are provided in Additional file 3. The raw and normalized RNA-seq counts are provided as processed data in ArrayExpress (see below for dataset identifiers).

### TMT proteomics

MEF cultures for five biological replicates of each genotype were washed with ice cold PBS. Cells were lysed in 200 μl of 0.1 M TEAB, 0.1% sodium dodecyl sulphate (SDS) at 90 °C for 10 min, followed by tip sonication. Total protein was quantified using a Bradford assay (Bio-Rad, Quick Start) according to the manufacturer’s instructions. We reduced 90 μg of protein per sample by the addition of 2 μl 50 mM tris-2-carboxyethyl phosphine (ΤCEP) for 60 min at 60 °C followed by cysteine blocking for 10 min at room temperature using 1 μl 200 mM methyl methanethiosulfonate (MMTS). Trypsin digestion (protein/trypsin ratio 30:1) was performed overnight at 37 °C. Peptides were labeled using the TMT 10-plex reagents (Thermo Scientific). The TMT mixture was then basic reverse phase (bRP) fractionated on a Dionex Ultimate 3000 system at high pH using the X-Bridge C18 column (3.5 μm 2.1 × 150 mm, Waters).

Fractions were analyzed on a Dionex Ultimate 3000 UHPLC system coupled with the nano-ESI Fusion Lumos (Thermo Scientific). Samples were loaded on the Acclaim PepMap 100, 100 μm × 2 cm C18, 5 μm, 100-Å trapping column with the ulPickUp injection method using the loading pump at 5 μl/min flow rate for 10 min. For the peptide separation the EASY-Spray analytical column 75 μm × 25 cm, C18, 2μm, 100 Ȧ was used for multi-step gradient elution. Mobile phase A was composed of 2% acetonitrile, 0.1% formic acid and mobile phase B was composed of 80% acetonitrile, 0.1% formic acid. The Lumos was operated in a data-dependent mode for both MS2 and SPS-MS3 methods. The full scan was performed in the Orbitrap in the range of 380–1500 m/z at 120 K resolution and the MS2 scan was performed in the ion trap with collision energy 35%. Peptides were isolated in the quadrupole with isolation window 0.7 Th. The ten most intense fragments were selected for Synchronous Precursor Selection (SPS) HCD-MS3 analysis with MS2 isolation window 2.0 Th. The HCD collision energy was set at 55% and the detection was performed with orbitrap resolution 60 k and in scan range 100–400.

### Proteomic data processing and analysis

Raw data were processed with the Sequest HT search engine on Proteome Discoverer 2.1 software. All spectra were searched against a UniProtKB/Swiss-Prot fasta file containing 16,915 reviewed mouse entries. The parameters for the SequestHT node were as follows: Precursor Mass Tolerance 20 ppm, Fragment Mass Tolerance 0.5 Da, Dynamic Modifications were Oxidation of M (+ 15.995 Da), Deamidation of N, Q (+ 0.984 Da) and Static Modifications were TMT6plex at any N-Terminus K, (+ 229.163 Da) and Methylthio at C (+ 45.988). The consensus workflow included S/N calculation for TMT intensities as previously described [74], and the level of confidence for peptide identifications was estimated using the Percolator node.

Peptide intensity data were quantile normalized and summarized into protein-level counts by summing the intensity values for all peptides for a given protein. Samples were inspected via hierarchical clustering and PCA to identify outliers of low quality; these were removed from downstream analyses (wild-type replicates 2 and 3 and *Ctcf* hemizygous replicates 1 and 3). Limma 3.33.14 [73] was used to assess differential protein expression between the genotypes, controlling for batch effects (sample collection time). Detailed results are provided in Additional file 4.

### Hi-C

MEF cultures for three biological replicates of each genotype were fixed in DMEM containing 2% fresh formaldehyde and incubated at room temperature for 10 min, quenched with 1 M glycine for 5 min, and washed twice with ice cold PBS. The fixed cells were lifted off the plate, pelleted by centrifugation, and flash-frozen at −80 °C. Cross-linked cells were lysed according to published protocols [8, 75, 76], followed by chromatin HindIII digestion, biotinylation, ligation, proteinase K treatment, DNA purification, sonication, end repair, biotin pull-down, adapter ligation, and PCR amplification. Library fragment size measurement and quantification were performed using a 2100 Bioanalyzer (Agilent). Pooled libraries were sequenced on a HiSeq4000 according to the manufacturer’s instructions to produce paired-end 150 bp reads.

### Hi-C data processing and analysis

Each sample was sequenced to a mean depth of ~ 179 million paired-end reads, totaling over a billion read pairs for the complete dataset. Data were mapped and QCed with HiCUP 0.5.8 [77] and bowtie2 2.2.8 [78], using the GRCm38 mouse reference genome. Over 60% of all read pairs were properly mapped and paired; from these, over 85% were valid pairs and the uniqueness percentage after de-duplication was ~ 70%. The BAM files produced by HiCUP, which contain only valid, non-redundant read pairs, were used for downstream analyses.

The HiCUP output BAM files were converted to a format compatible with HOMER by using the hicup2homer utility. Using HOMER [79], tag directories were created from the merged data of the three wild-type or *Ctcf* hemizygous samples. The correlation between the interaction profiles of the two genotypes was calculated with the getHiCcorrDiff.pl script, using a resolution of 100 kb and a super-resolution of 150 kb.

To identify chromatin loop interactions, the analyzeHiC program from HOMER [79] was used on the merged data from all six replicates; this ensures that the definition of significant interactions is agnostic to the genotype, allowing us to subsequently perform differential analysis between the wild-type and hemizygous profiles without compromising FDR control [80]. We supplied analyzeHiC with the options –res 20,000 –interactions –nomatrix –maxDist 10000000 –minDist 5000 –center. Differential analysis on the identified loops was performed using diffHic [81], on the set of interactions reported by HOMER with an FDR lower than 0.05, and restricted to the autosomes. The HiCUP output BAM files were processed with the preparePairs function, keeping data for each replicate separate; any fragments mapping against the blacklisted regions used for the ChIP-seq analyses (see above) were discarded. Then, the function connectCounts was used to count the number of fragments mapping specifically to the loci involved in the loop interactions. Only interactions that had more than 20 average counts per million (90,704 loops) were used for differential testing. Data were normalized for depth of sequencing by providing the library sizes of the complete dataset. Differential testing was performed controlling for batch effects (sample collection time), and the resulting *p* values were corrected for multiple testing by the Benjamini and Hoechberg procedure. Finally, we used this ranked list to test whether looping interactions overlapping with differentially expressed genes (plus 5 kb on either side) or differentially bound CTCF sites were enriched at the top of the list, with the function geneSetTest (Wilcoxon signed rank test) from the limma package.

### Definition of gene–enhancer pairs

To determine if the enhancers that are likely to regulate differentially expressed genes are changing concordantly with gene expression, we retrieved all putative enhancer peaks (defined as H3K27ac peaks that did not overlap with H3K4me3 peaks) that were linked to a differentially expressed gene via a significant interaction in the Hi-C data (see above). For each of the 296 dysregulated genes, 261 had at least one and up to 65 linked enhancers (median = 8). Only a subset of these gene–enhancer pairs are likely to be bona fide regulatory interactions. To increase our signal-to-noise ratio, we reasoned that we could use the paired nature of our datasets to infer correlation between the RNA expression levels and H3K27ac abundance, since both measurements were performed in the same MEF cultures. Thus, for each gene–enhancer pair we calculated the Pearson correlation coefficient between the RNA-seq and ChIP-seq normalized counts for the five replicates that had successful libraries for both methodologies. We then retained the gene–enhancer pair with the highest correlation value for each dysregulated gene. Figure 5a was plotted with these pairings; for genes with no linked enhancer, the corresponding row in the heatmap has been left blank.

To generate the heatmaps shown in Fig. 5a we defined 1kb windows centered either at the transcription start site (as defined in Ensembl v84) or the midpoint of the H3K27ac peak, extending 17 kb up and downstream. The number of sequencing reads mapping to such windows in the histone ChIP-seq data were obtained with BEDTools v2.24.0, command *intersect –c* [82]. The counts for each sample were normalized to account for the total depth of sequencing and then aggregated into 5 kb bins. For each 5 kb bin the average abundance across all replicates was used, and the log_2_ fold change between the genotypes was plotted.

### Mouse liver and tumor samples

Male C3H/HeOuJ mice, which are susceptible to spontaneous liver neoplasms [49], were aged until they showed clinical signs of tumor development (up to 76 weeks old). Liver tissue samples from four young, tumor-free mice were collected for control experiments. Liver tumors (*n* = 25) and liver tissue samples were snap frozen in liquid nitrogen and total RNA was extracted using the AllPrep 96 DNA/RNA Kit (Qiagen) according to the manufacturer’s instructions. Library preparation and sequencing were performed as described before. Sample information is provided in Additional file 5.

### Mouse tumor data analysis

The RNA-seq data from mouse liver and tumor samples was processed as detailed above but using the C3H/HeJ genome as a reference (ftp://ftp.ensembl.org/pub/release-89/fasta/mus_musculus_c3hhej/dna/Mus_musculus_c3hhej.C3H_HeJ_v1.dna_sm.toplevel.fa.gz) [83]. To test for differential expression between the normal liver and tumor samples we used DESeq2 [72] and genes were considered significantly differentially expressed if their adjusted *p* value was lower than 0.05 (FDR < 5%).

To compare to the list of differentially expressed genes in the *Ctcf* hemizygous MEFs, we matched genes by their official gene name. Genes that were significantly differentially expressed in both datasets were deemed concordant if they were up or downregulated both in the *Ctcf* hemizygous MEFs and in the tumors, compared to their respective controls. Gene set enrichment analysis was performed as detailed previously, using only the set of concordant differentially expressed genes as the test set. Detailed results of both the differential expression and gene set enrichment analyses are provided in Additional file 5. The raw and normalized RNA-seq counts are provided as processed data in ArrayExpress (see below for dataset identifiers).

### The Cancer Genome Atlas data analysis

To compare the set of dysregulated genes in the *Ctcf* hemizygous MEFs to alterations in the transcriptomes of human cancers, we mined The Cancer Genome Atlas PanCanAtlas to obtain a list of uterine and breast tumor samples with identified missense, frameshift, or stop-gain mutations in *CTCF*. The identifiers of all the samples used are detailed in Additional file 5. We collected the RNA-seq raw counts for these samples, along with all available control normal uterine and breast tissue samples. The gene annotation used was from https://www.gencodegenes.org/releases/22.html. We used DESeq2 [72] to normalize and test for differential expression between the tumor and control samples, for each tissue separately.

To compare these results to the genes altered in the *Ctcf* hemizygous MEFs, we obtained the orthology relationships between the human and mouse genome using Ensembl version 84 [70] and restricted our analysis to one-to-one orthologs. Genes that were significantly differentially expressed in both datasets were deemed concordant if they were up- or down-regulated both in the *Ctcf* hemizygous MEFs and in the tumors, compared to controls. Detailed results of the differential expression analyses are provided in Additional file 5.

## Additional files

Additional file 1: Validation of *Ctcf* deletion. Quantification of *Ctcf* deletion by qRT-PCR and quantitative western blot experiments on wild-type and *Ctcf* ^+/−^ MEFs. Format: xls. File size: 61 KB. (XLS 40 kb)

Additional file 2: Differential binding results of ChIP-seq datasets. Results from the differential binding analyses of the CTCF ChIP-seq dataset using csaw, and of the H3K4me3 and H3K27ac datasets using DiffBind. Format: xls. File size: 20.5 MB. (XLS 20040 kb)

Additional file 3: Differential expression results of RNA-seq data. Results from the differential expression analysis of the RNA-seq dataset using DESeq2. Also, results from the gene set enrichment analyses for both Gene Ontology sets and KEGG pathways. Format: xls. File size: 13.5 MB. (XLS 13174 kb)

Additional file 4: Proteome quantification. Normalized protein abundances from the TMT proteomics dataset along with the results from the differential abundance analysis. Format: xls. File size: 2.9 MB. (XLS 2840 kb)

Additional file 5: Mouse and human tumor metadata and differential expression results of RNA-seq data. Sample information regarding all mouse and human tumor samples included in analyses. Results from the differential expression analysis of mouse liver tumors and human uterine and breast cancer dataset (performed using DESeq2). Gene set enrichment analysis for mouse concordant genes. Intersection results of MEF and tumor DE analyses. Format: xls. File size: 20.7 MB. (XLS 20148 kb)
